# Enhancing Unsupervised Multi-Source Domain Adaptation for Person Re-Identification via Mixture of Experts and Graph-Based Relation

**DOI:** 10.3390/s26123968

**Published:** 2026-06-22

**Authors:** Hao Li, Yuyang Feng, Xin Zhao, Xuan Li, Tao Zhang

**Affiliations:** 1 School of Electrical and Information Engineering, Tianjin University, Tianjin 300072, China; tjdxlihao@tju.edu.cn (H.L.); fengyuyang@tju.edu.cn (Y.F.); zhaoxin_16@tju.edu.cn (X.Z.); 2 College of Automotive Engineering, Tianjin Vocational Institute, Tianjin 300410, China; 3 School of Electrical Engineering and Automation, Tianjin University of Technology, Tianjin 300384, China; lixuantju@tju.edu.cn

**Keywords:** person re-identification, Mixture of Experts, domain-specific style information, adjacency matrix

## Abstract

Person re-identification (re-ID) aims to match pedestrian images across disjoint camera views. Existing multi-source unsupervised domain adaptation (UDA) re-ID methods still face two critical issues: they fail to effectively balance domain-invariant feature learning and domain-specific style preservation and cannot adequately model the implicit correlations among diverse source domains, resulting in limited cross-domain generalization performance. To address these challenges, this paper proposes a novel multi-source UDA re-ID framework equipped with a Mixture of Experts feature extraction (MEFE) network and a Graph-Based Relation (GBR) module. Specifically, the MEFE network integrates mixed Instance and Batch Normalization (MIBN) to extract robust domain-invariant features, while the embedded domain-specific style information (DSI) module compensates for lost domain-specific style details at the feature level. Furthermore, the cascaded Graph Attention and Graph Convolution Networks (GATs/GCNs) in the GBR module adaptively explore implicit feature correlations and achieve effective multi-source feature fusion. Center maximum mean discrepancy loss is adopted to further reduce cross-domain distribution discrepancies. Extensive experiments on large-scale datasets demonstrate that the proposed method achieves state-of-the-art performance and substantially outperforms mainstream UDA re-ID approaches.

## 1. Introduction

Person re-identification (re-ID) aims at matching persons of the same identity across different camera views. Though supervised re-ID methods have achieved great performance, the performance dramatically drops when deploying the well-trained model from source domain to target domain because of domain shifts [1,2,3]. To alleviate this problem, recent efforts have studied unsupervised domain adaptation (UDA) methods, which train model on source domain by labeled data and utilize unlabeled target data to finetune. Most existing UDA methods focus on single-source UDA, where only one labeled source domain is available [4,5,6]. Multi-source UDA provides multiple labeled datasets of different source domains; the ground-truth labels of the source domain and pseudo-labels of the target are collected into a hybrid dataset to train a single model. However, such direct combining of datasets brings limited improvement or even a negative impact. Recent methods typically overlook the feature diversity among multiple sources [7,8] and fail to exploit the complementary knowledge from heterogeneous domains [9,10]. Meanwhile, they cannot maintain label consistency and structure consistency across distinct source domains [11,12], and ignore the relational distribution alignment between multiple sources and the target [13,14]. Since the environmental conditions vary drastically in different domains, the inconsistency caused by large domain gaps among different domains limits performance improvement. Such a method learns a common feature space for different source domains, ignoring individual source domains’ discriminative information and the relationship among source domains and the target domain.

**Figure 1:**
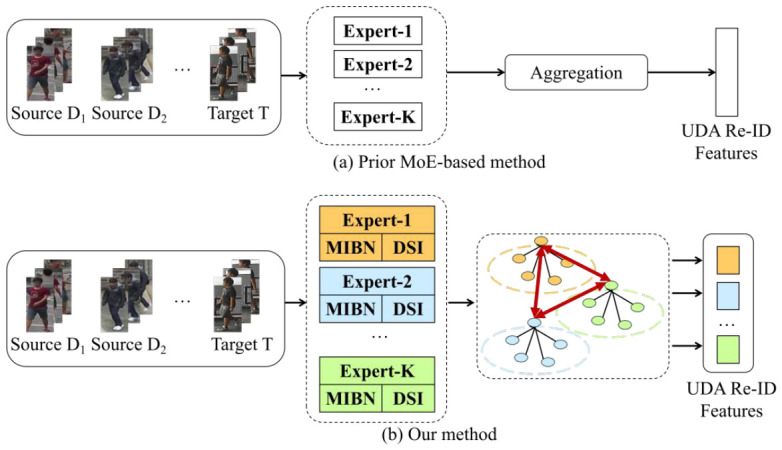
Differences between our method and the conventional method of unsupervised multi-source domain adaptation person re-ID. (**a**) Conventional methods adopt a multiple domain-specific network for multi-source domain. (**b**) Our method leverages one general branch sharing all the parameters except for the normalization layers.

We try to solve this problem from two perspectives, i.e., diversity relations and consistent relations. For diversity relations, some current works have been devoted to the Mixture of Experts (MoE) [15,16] method to improve the model’s robustness by adopting multiple domain-specific networks for each source domain to obtain the individual source domain’s discriminative information. These methods can exploit the diversified characteristics of each source domain and provide rich complementary information but will result in a large model size. Inspired by MoE, we propose an approach called the Mixture of Experts feature extraction (MEFE) network for UDA of person re-ID, which does not adopt multiple domain-specific networks; instead, it applies one general branch sharing all the parameters except for the normalization layers to avoid an excessively large model [17], as shown in Figure 1. Batch Normalization (BN) statistics are computed for capturing discriminative features and Instance Normalization (IN) statistics for eliminating domain-specific information.

It is demonstrated that the combination of BN and IN in a concise manner is more powerful than only BN to improve generalization and discrimination capabilities of model [18]. Based on this, we introduce a mixture of Instance Normalization and Batch Normalization (MIBN) into multiple experts to extract invariant features across domains, which combines BN layers and Instance Batch Normalization type-a (IBN-a) layers to compensate the missing domain-invariant information across domains in the feature level.

Studies on style transfer [19,20,21] suggest that the style information for each image can be represented via means and standard deviations computed within each channel of feature. Moreover, to exploit the diversified characteristics of each source domain and provide rich complementary information further, we propose a new feature stylization approach (DSI). It compensates meaningful style information to BN and IN parameters, providing more complementary information to improve the features’ generalizability on target domain.

In consistent relations fields, it is crucial to minimize the gap between source domain and target domain. Thus, some works maintain a common space for both domain-invariant and domain-specific features [22] and some introduce high-dimensional intermediate latent space to enable source domain and target domain share similar distribution [23]. Unfortunately, these previous works overlook the relationship between source domain and target domain. To tackle this issue above, some domain-generalizable (DG) re-ID methods introduce MoE to improve the generalization of models by integrating multiple domain-specific expert networks, but multiple sub-networks will bring heavy computational burden [24]. GCN-based re-ID methods [25] can enhance feature representation by establishing a mathematical relation model, as well as reducing domain gaps among multiple source domains and the target domain. Based on previous works, we design a Graph-Based Relation (GBR) module to connect multiple domain features. Owing to domain gaps, the connection weight of different source domains node to target domain node is unequal, but similar domain should be assigned greater weight. Moreover, intra-domain nodes that live nearby domain center should be denoted to larger adjacency matrix. Considering these factors, our GBR module cascades Graph Attention Networks (GATs) and Graph Convolution Networks (GCNs); GAT is used to integrate multiple domain centers adaptively by introducing an attention mechanism. For intra-domain nodes, the adjacency matrix is computed by a Gaussian similarity function, encouraging the nodes nearby domain center to contribute greater weight.

Our major contributions can be summarized as follows:

(1) We propose a multi-source learning framework for UDA of person re-ID based on mutual learning by exploiting the diversity and consistent relations of multiple source domains and the target domain.
(2) We develop a Mixture of Experts feature extraction (MEFE) network that adopts one general branch sharing all the parameters except for the normalization layers to learn domain-specific information. A mixture of Instance Normalization and Batch Normalization (MIBN) that combines BN layers and IBN-a layers is introduced into multiple experts to extract invariant features across domains, and a domain-specific style information (DSI) module is embedded into multiple experts to compensate the missing domain-specific style information at the feature level.
(3) We design a Graph-Based Relation (GBR) module to integrate multiple domain features adaptively via cascading Graph Attention Networks (GATs) and Graph Convolution Networks (GCNs), and a Gaussian similarity function is utilized to exploit the implicit relation among features to construct the normalized relation matrix in the GBR module.
(4) Extensive experiments demonstrate that our method outperforms existing UDA person re-ID approaches on large-scale and achieves state-of-the-art results.

## 2. Related Work

### 2.1. Unsupervised Domain Adaptation for Person Re-ID

The UDA method aims to train models on an annotated source domain by labeled data and transfer knowledge from the labeled source domain to the unlabeled target domain. The giant domain gap between source domain and target domain would lead to deteriorating performance. The UDA methods for person re-ID can be divided into two categories: Generative Adversarial Network (GAN)-based methods and clustering-based methods. GAN-based methods preserve the original identities of the source images and transform source domain images to match the target domain style. For clustering-based methods, the performance of models depends on the quality of pseudo-labels, so many studies have been devoted to improving pseudo-label quality. Clustering-based methods are involved in three fields, the joint learning-based method, pseudo-label noise-based method and memory bank-based method. Joint learning-based methods focus on exploring the potential representation of global and local parts [26,27,28] to obtain up-to-date features. Pseudo-label noise-based methods aim to alleviate pseudo-label noise by bringing soft pseudo or employing a mutual teacher–student framework to refine the noisy pseudo-labels [29,30]. Memory bank-based methods learn invariant features [31] using a memory bank and calculate the contrastive loss of positive sample and negative sample pairs in the memory bank. UDA methods rely on the transferable knowledge learned from the source domain, but the discriminative information of the target domain may not be fully explored.

### 2.2. MoE-Based Method

For most domain-generalizable person re-ID methods, multiple source domains are directly fed to the model to learn discriminative features without considering the relevance among them. MoE assigns a branch network expert to each source domain to extract domain-specific features and then aggregates multiple experts to unseen domains [24]. It has proved its superiority in image recognition and machine translation. Some methods combine the experts with a learning voting network to improve generalization ability [15]. However, multiple branches of the source domain increase the complexity of the model and bring additional computational costs. To tackle this problem, DSBN adopts one general branch sharing all the parameters except for the BN layers to learn domain-specific information [8], which simplifies the model and improves the stability of model optimization. Furthermore, RDSBN adopts a rectification procedure to enhance identity-related information and make features more discriminative [32]. However, the above methods do not consider enhancing the capacity of extracting the domain-specific features of each expert.

### 2.3. Feature Normalization

It has been proven that Batch Normalization (BN) can extract domain-specific discriminative features and Instance Normalization (IN) can capture domain-invariant information by losing domain-specific information. IBN-Net combines BN and IN in two ways to increase generalization and discrimination capability of network [18]. Chen denotes the mean and standard deviation of the feature in channel dimension as the style information [33]. Dynamically Transformed Instance Normalization (DTIN) is proposed to adapt to individual domains [34]. The parameters of BN layers vary in different domains; previous methods calculate the style information through normalization without considering the style difference of multiple domains [20]. To alleviate this problem, domain-specific BN is exploited, sharing all the parameters except for the Batch Normalization (BN) layers. DSL performs style transfer by mixing style information of different domains extracted from expert networks [35]. These approaches improve accuracy by choosing proper normalization or compensating for the missing information.

### 2.4. Graph Convolution Network

A GCN usually is used to deal with graph-structured problems and demonstrates impressive performance in the task of computer vision [36]. Several works model structural relations between key patches to capture structural relations by leveraging a GCN [37]. Different from all the methods mentioned above, some approaches employ a simple GCN algorithm to generate discriminative fusion features to improve the quality of pseudo-labels [38]. Simultaneously, some methods improve accuracy by exploiting the GCN in multiple domains to reduce domain gaps [32]. Nevertheless, these works only focus on utilizing relation information but do not explicitly exploit the feature of the edge between two nodes on the graph adaptively.

## 3. Method

In this work, we propose a novelty unsupervised multi-source domain adaptation framework for person re-ID. The framework of our approach consists of two parts: a Mixture of Experts (MoE) feature extraction network, and a multi-domain fusion module. Firstly, for the former network, we aim to train a group of normalization experts that can extract discriminative domain-specific and domain-invariant features without losing domain style information from their individual domains. Secondly, the latter multi-domain fusion module is used to adaptively integrate all the source experts’ features into aggregated features to reduce domain gaps. We adopt a GAT to learn domain-level weight adaptively between multiple domains and employ a Gaussian similarity function to compute feature-level weight among features in the GCN.

### 3.1. Overview

**Figure 2:**
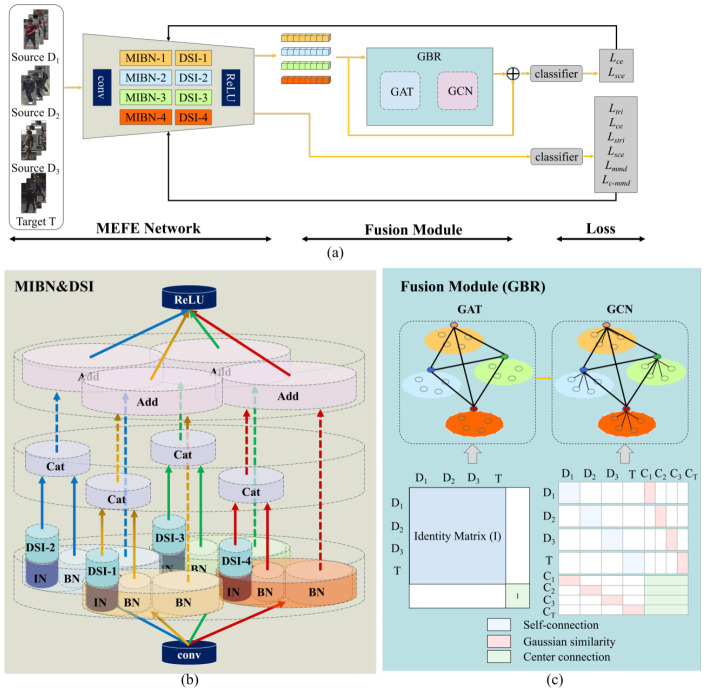
The training strategy of our method. (**a**) The feature extraction network of our method, which comprises MEFE network and Fusion Module. (**b**) The normalization layer in MEFE network, which combines MBIN and DSI for each expert. (**c**) The structure of fusion module, cascading Graph Attention Networks (GATs) and Graph Convolution Networks (GCNs) to integrate multiple domain features adaptively.

The framework of our proposed method for multi-source unsupervised domain adaptation re-ID is illustrated in Figure 2. The labeled source domain datasets are denoted as $S=\left\{\left\{X_{1},Y_{1}\right\},\left\{X_{2},Y_{2}\right\},\left\{X_{3},Y_{3}\right\}\right\}$, and the unlabeled target domain is recorded as $T=\left\{X_{4},Y_{4}\right\}$. X_i_ and Y_i_ represent the sample images and ground-truth labels of the i-th domain, respectively. We take sample images of three source domains and one target domain as input of model. The backbone extract features of four domains using a sharing branch except for the normalization layers. For a mini-batch, the normalization layer sets an MIBN for each domain and then add DSI for each domain. The output features are fed into multi-domain fusion module, and weighted aggregated features are obtained by its neighbor domains and neighbor features successively. The details of the backbone module and multi-domain fusion module are described in Figure 2. The training process is implemented based on the mutual mean teaching strategy from [29], which exhibits superior capability in re-ID applications. Our collaborative training system includes four identical networks: Net A, Net B, Mean Net A and Mean Net B. Hard classification loss and hard triplet loss are computed via Net A and Net B. In order to reduce mutual bias during cooperation, we derive soft classification loss and soft triplet loss from the corresponding averaged models (Mean Net A and Mean Net B). The detailed workflow of our method is summarized in Algorithm 1.
**Algorithm 1:** Unsupervised multi-source domain adaptation method for person re-identification via mixture of experts and graph-based relation**Input**: Network $F(\cdot |\theta_{1})$, $F(\cdot |\theta_{2})$, $F(\cdot |E^{\left(T\right)}(\theta_{1}))$, $F(\cdot |E^{\left(T\right)}(\theta_{2}))$ pseudo-labeled training dataset. $x_{i}^{t}$ and $x_{i}^{\prime t}$
**Output**: Updated $F(\cdot |\theta_{1})$, $F(\cdot |\theta_{2})$, $F(\cdot |E^{\left(T\right)}(\theta_{1}))$, $F(\cdot |E^{\left(T\right)}(\theta_{2}))$1: **for** epoch in range (epochs):2:         Extract features of sample instances by MEFE of Net A, Net B, Mean Net A and Mean Net B3:         Extract features of sample instances by GBR of Net A, Net B, Mean Net A and Mean Net B4:             Clustering and generating $T_{i}(\theta_{1})$, $T_{i}(\theta_{2})$, $T_{i}(E^{\left(T\right)}[\theta_{1}])$, $T_{i}(E^{\left(T\right)}[\theta_{2}])$ and $C_{1}^{t}(F(x_{i}^{t}|\theta_{1}))$, $C_{2}^{t}(F(x_{i}^{\prime t}|\theta_{2}))$, $C_{1}^{t}(F(x_{i}^{t}|E^{\left(T\right)}[\theta_{1}]))$, $C_{2}^{t}(F(x_{i}^{\prime t}|E^{\left(T\right)}[\theta_{2}]))$
5:         Compute the hard classification loss by Equation (8)6:         Compute the soft classification loss by Equations (9) and (10)7:         Compute the hard triplet loss by Equation (12)8:         Compute the soft triplet loss by Equations (14) and (15)9:         Compute the maximum mean discrepancy (mmd) loss by Equation (16)10:       Compute the center maximum mean discrepancy (c-mmd) loss by Equation (17)11:       Compute overall loss by Equation (18)12:       Backward to update $F(\cdot |\theta_{1})$, $F(\cdot |\theta_{2})$
13:       Update $F(\cdot |E^{\left(T\right)}(\theta_{1}))$, $F(\cdot |E^{\left(T\right)}(\theta_{2}))$ by Equation (11)14:**end for**

### 3.2. Mixture of Experts Feature Extraction

As mentioned in [11], exploiting the complementary information of discriminative experts enables the model to improve the generalization on the target domain. For the backbone structure, we select ResNet-50 and adjust the stride of res_conv5x to 1. Such a configuration contributes to the excellent performance of the model in person re-identification tasks. We apply MIBN for each domain after the first convolution layer in the residual path. MIBN has two branches, one branch applies BN for half channels and IN for the others, and the other branch feeds features to BN layers. The features of two branches are added together. MIBN is utilized in the first three groups (conv2_x-conv4_x) and leaves the fourth group (conv5_x) as before for each domain feature. We calculate the mean and standard deviation of each domain feature in the channel dimension as style information after each IBN-branch, as shown in Figure 2b. Referring to AdaIN [15], we replace the scale and shift parameters in Equation (1) with the feature statistics of mean and standard deviation to learn style information.

(1) $$AdaIN(x)=\sigma (y)\frac{x-\mu (x)}{\sigma (x)}+\mu (y)$$

where μ (x)/μ (y), σ(x)/σ(y) are the mean and standard deviation computed across the spatial dimension within each channel of each instance (tensor).

#### 3.2.1. Normalization Layer of Expert Branch

The prior MoE-based algorithms set one expert branch for each domain to learn different domains, which have good domain generalization. However, such models suffer from a large model size with increasing domains. To address this problem, Xu et al. [20] set specific BN layers for different domains in the model. We take mixed IN and BN layers named MIBN for each domain and employ one general branch sharing all the parameters except for the batch normalization layers. MIBN has two branches, one branch applies BN for half the channels and IN for the others, and the other branch feeds features to BN layers. Finally, the features of the two branches are added together. We take sample images of three source domains and one target domain as the input of the model. The general branch ensures the discriminability of the sample in the feature space, and the expert MIBN carries obvious domain style information. Meanwhile, our multi-source domain has rich labeled data, which will improve the generalization of model.

#### 3.2.2. Domain-Specific Style Information Module

Since the IN layer will eliminate style differences, we explore the DSI module following the IN branch to compensate the missing domain-specific style information at the domain level. AdaIN simply replaces the scale and shift parameters with the mean value and standard deviation of style input y to achieve style transfer from x to y. According to AdaIN, the input features multiply standard deviation and plus the mean value of style input to complement the missing domain-specific style information.

### 3.3. Multi-Domain Feature Fusion Adaptive Learning

In this section, we design a multi-domain fusion module to integrate multiple domain features adaptively via cascading Graph Attention Networks (GATs) and Graph Convolution Networks (GCNs). The GAT is applied to connect domains and the GCN is used to fuse features of the same domain. Specifically, as shown in Figure 2c, each input feature vector acts as a node in the graph, and an edge records a domain similarity or an individual feature similarity between two nodes. The similarity of domains is different; the node should have different priorities. Initially, all domain center nodes have the same priority; the priority of each center node is changed through the GAT. In terms of the domain center to individual features, a Gaussian similarity function is utilized to exploit the implicit relation among features to construct the normalized relation matrix in the GCN.

#### 3.3.1. Domain-Level Weight Adaptive Learning

The GAT has a hidden layer; the nodes of the input are the centers of each domain. Domain centers are obtained by calculating the average of all features. There are 3 source domains and 1 target domain in our approach. Assuming the batch size is n, there are in total 3xn feature vectors and 3 domain center feature vectors. Each edge $e_{ij}$ represents a connection between feature vectors, and the corresponding weight value $a_{ij}$ indicates the connection strength between them. For inter-domain edges, we define the domain center nodes connect each other and the rest nodes have no edges. Initially, all domain centers nodes have the same priority; the weight value of each edge is obtained through the GAT from the second loop. The GAT learns to assign importance to attention coefficients to each neighbor of a node. This allows for the model to focus on more relevant neighbors and filter out noise.

Given a training set S and a testing set T, we construct a graph G=(H,E), where $H\left(\left|H\right|=N\right)$ and E are sets of nodes and edges, respectively. Each node $h_{i}$ represents a person feature vector, and each edge $e_{ij}$ represents a connection between $h_{i}$ and $h_{j}$. The corresponding weight value $a_{ij}$ indicates the connection strength between them. Here, we define two kinds of edges: the domain edge to measure the domain adjacency between two domains, and the individual feature edge to measure the individual feature similarity between them. The calculation details are as follows:

(2) $$h_{i}^{\left(l+1\right)}=\sum_{j\in N(i)}a_{ij}W^{\left(l\right)}h_{j}^{\left(l\right)}$$

(3) $$a_{ij}=\mathrm{soft}\;\max_{j}(e_{ij})=\frac{\exp(e_{ij})}{\sum_{k\in N_{i}}\exp(e_{ik})}$$

(4) $$e_{ij}=a(W\vec{h_{i}},W\vec{h_{j}})$$

where $h_{i}$ and $h_{j}$ are two domain center nodes; a indicates self-attention mechanism. W represents the weight matrix; $a_{ij}$ is a learnable weight value that is computed by the attention mechanism.

#### 3.3.2. Instance-Level Weight Adaptive Learning

In the GCN, all domain center nodes have the same priority, the edge values between domain center nodes and feature nodes are calculated by Gaussian similarity function for intra-domain edges, which assigns weights between vectors based on their pairwise similarity. It quantifies the strength of association or influence between feature nodes and its domain center. The Gaussian similarity function is summarized as

(5) $$A_{i,j}^{\left(l\right)}{=a}_{ij}=\exp\left(-\frac{d(h_{i},h_{j})}{2\sigma^{2}}\right)$$

where $d\left(h_{i},h_{j}\right)$ is the distance measure and σ is the length scale parameter. We choose Euclidean distance as feature distance and assign 2 to σ. The neighborhood structure behaves differently with respect to various σ, which means that it needs to carefully select the optimal σ for the best performance of label propagation.

(6) $$H^{\left(l\right)}=\rho \left(\tilde{A^{\left(l\right)}H^{\left(l-1\right)}W^{\left(l\right)}}\right)$$

(7) $$\overset{\wedge }{H}=H^{\left(0\right)}+H^{\left(2\right)}$$

where W represents weight matrix, ρ denotes a non-linear function that is LeakyReLU in our method. $H^{\left(0\right)}$ is defined as the input features, $H^{\left(2\right)}$ is the output features after the GAT and GCN. $\overset{\wedge }{H}$ represents fused features, which adds $H^{\left(0\right)}$ and $H^{\left(2\right)}$.

### 3.4. Loss Function

The employment of four fundamental re-ID loss functions suffices for achieving optimal performance, namely the hard triplet loss, hard classification loss, soft triplet loss, and soft classification loss. These four losses are applied after MEFE network, while hard classification loss of GBR and soft classification loss of GBR are applied after the multi-domain fusion module. The triplet loss is used to pull similar samples closer and push different samples apart before regularization, which can improve the model’s classification accuracy. Cross-entropy loss can measure the model’s prediction accuracy for each class. We adopt mmd loss to reduce the distribution discrepancy between the source domain and target domain, improving the model’s generalization performance on the target domain. At the same time, feature distributions of different classes may exhibit large intra-class variance and inter-class overlap, so we introduce c-mmd loss addresses to enforce feature center of same-class samples to align closely between source and target domains, reducing intra-class discrepancy.

The hard classification loss can calculate as

(8) $$L_{id}^{t}(\theta_{1})=\frac{1}{N_{t}}\sum_{i=1}^{Nt}L_{\mathrm{ce}}(C_{1}^{t}(F(x_{i}^{t}|\theta_{1})),{\overset{\sim t}{y\prime }}_{1})$$

where θ represents the network parameters, $C_{1}^{t}(F(x_{i}^{t}|\theta_{1}))$ denotes the pseudo-label confidence of each branch, and $C_{1}^{t}$ represents the target-domain classifier of two networks. $L_{\mathrm{ce}}$ represents the cross-entropy classification, ${\overset{\sim t}{y\prime }}_{1}$ is the pseudo-labels generated by clustering.

The soft classification loss is formulated as

(9) $$L_{sid}^{t}(\theta_{1}|\theta_{2})=-\frac{1}{N_{t}}\sum_{i=1}^{N_{t}}(C_{2}^{t}(F(x\prime_{i}^{t}|E^{\left(T\right)}[\theta_{2}])).\log C_{1}^{t}(F(x_{i}^{t}|\theta_{1})))$$

(10) $$L_{sid}^{t}(\theta_{2}|\theta_{1})=-\frac{1}{N_{t}}\sum_{i=1}^{N_{t}}(C_{1}^{t}(F(x_{i}^{t}|E^{\left(T\right)}[\theta_{1}])).\log C_{2}^{t}(F(x\prime_{i}^{t}|\theta_{2})))$$

where

(11) $$E^{\left(T\right)}[\theta ]=\alpha \;E^{\left(T-1\right)}[\theta ]+(1-\alpha )\theta \;\theta \in \left(\theta_{1},\theta_{2}\right)$$

The parameters of the time-average model are updated with the last period of parameters of itself and the current period original corresponding model parameters.

Hard softmax–triplet loss and soft softmax–triplet loss are proposed to optimize triplet loss with soft pseudo-labels. The hard softmax–triplet loss is defined as

(12) $$L_{\mathrm{tri}}^{t}\left(\theta \right)=\frac{1}{N_{t}}\sum_{i=1}^{N_{t}}L_{bce}\left(T_{i}\left(\theta_{1}\right),1\right)$$

where

(13) $$T_{i}\left(\theta_{1\mathrm{or}2}\right)=\frac{\exp({\left[F(x_{i}^{t}|\theta_{1\mathrm{or}2})\right]}^{T}F(x_{i,n}^{t}|\theta_{1\mathrm{or}2}))}{\exp({\left[F(x_{i}^{t}|\theta_{1\mathrm{or}2})\right]}^{T}F(x_{i,p}^{t}|\theta_{1\mathrm{or}2}))+\exp({\left[F(x_{i}^{t}|\theta_{1\mathrm{or}2})\right]}^{T}F(x_{i,n}^{t}|\theta_{1\mathrm{or}2}))}$$

$L_{bce}(\cdot ,\cdot )$ is denoted as binary cross-entropy loss. The subscripts i, p and i, n are the hardest positive and negative samples of $x_{i}^{t}$ in the batch. Soft triplet labels of two networks are supervised by the other average model during mutual training, which is important for improving domain adaptation performance. The soft softmax–triplet loss is employed to enhance the final ranking performance, which is written as

(14) $$L_{stri}^{t}(\theta_{1}|\theta_{2})=\frac{1}{N}\sum_{i=1}^{N_{t}}L_{bce}(T_{i}\left(\theta_{1}\right),T_{i}(E^{\left(T\right)}[\theta_{2}]))$$

(15) $$L_{stri}^{t}(\theta_{2}|\theta_{1})=\frac{1}{N}\sum_{i=1}^{N_{t}}L_{bce}(T_{i}\left(\theta_{2}\right),T_{i}(E^{\left(T\right)}[\theta_{1}]))$$

The mmd and c-mmd loss are presented as

(16) $$L_{mmd}(S,T)=\frac{1}{k^{2}}\sum_{i=1}^{k}\sum_{j=1}^{k}\sigma (s_{i},s_{j})+\frac{1}{m^{2}}\sum_{i=1}^{m}\sum_{j=1}^{m}\sigma (t_{i},t_{j})-\frac{2}{km}\sum_{i=1}^{k}\sum_{j=1}^{m}\sigma (s_{i},t_{j})$$

(17) $$L_{c\text{-}mmd}(S_{c},T_{c})=\frac{1}{k^{2}}\sum_{i=1}^{k}\sum_{j=1}^{k}\sigma (s_{c_{i}},s_{c_{j}})+\frac{1}{m^{2}}\sum_{i=1}^{m}\sum_{j=1}^{m}\sigma (t_{c_{i}},t_{cj})-\frac{2}{km}\sum_{i=1}^{k}\sum_{j=1}^{m}\sigma (s_{c_{i}},t_{c_{j}})$$

where S and T represent the transformed source domain and target domain, respectively. k and m represent the number of samples in S and T, respectively. σ(⋅,⋅) is a Gaussian kernel. $s_{i}$ and $t_{i}$ are the ith samples from S and T, and $s_{c_{i}}$ and $t_{c_{i}}$ are the ith clustering center from S and T, respectively. The consistency of feature distribution between the transformed source domain and target domain determines whether the transformed source domain works well in the target domain. The features from different domains are often different; mmd is used to measure the difference of feature distribution in S and T.

The total loss function L(θ_1_, θ_2_) combines hard triplet loss, hard classification loss, soft triplet loss, and soft classification loss, which can be written as Equation (18):

(18) $$\begin{array}{l} L(\theta_{1},\theta_{2})=(1-\varsigma_{id}^{t})(L_{id}^{t}(\theta_{1})+L_{id}^{t}(\theta_{2})+L_{id}^{t}(\theta_{1\mathrm{GBR}})+L_{id}^{t}(\theta_{2GBR}))+(1-\varsigma_{stri}^{t})(L_{tri}^{t}(\theta_{1})+L_{tri}^{t}(\theta_{2}))\\ +\varsigma_{id}^{t}(L_{sid}^{t}(\theta_{1}|\theta_{2})+L_{sid}^{t}(\theta_{2}|\theta_{1})+L_{sid}^{t}(\theta_{1GBR}|\theta_{2GBR})+L_{sid}^{t}(\theta_{2GBR}|\theta_{1GBR}))\\ +\varsigma_{tri}^{t}(L_{stri}^{t}(\theta_{1}|\theta_{2})+L_{stri}^{t}(\theta_{2}|\theta_{1}))+L_{\mathrm{mmd}}+L_{c\text{-}mmd} \end{array}$$

## 4. Experiments

### 4.1. Datasets and Evaluation Protocol

We evaluate our methods on three widely-used person re-ID datasets, including Market-1501 [39], DukeMTMC-reID [40] and MSMT17 [41]. The Market-1501 contains 32,668 images of 1501 identifications from six cameras. The training set includes 12,936 images of 751 identities, the gallery set has 12,732 images of 750 identities and the query set consists of 3368 images of 750 identities.

DukeMTMC-reID has 36,411 images of 1812 identities from eight cameras, of which there are 16,522 images with 702 identities for training, 2228 images for query images and 17,661 images for the gallery.

MSMT17 stands as the largest and most challenging benchmark in person re-identification. Its training set comprises 1041 identities across 32,621 bounding boxes, while the test set contains 3060 identities with 93,820 annotated instances.

In evaluations, we use four widely used metrics to compare the performance of our method with the existing state of the art, i.e., Mean Average Precision (mAP) and Cumulative Matching Characteristic (CMC) at top1, top5, and top10.

### 4.2. Implementation Details

There are two stages, pre-training and mutual mean teaching adaptation training, in our experiments. All experiments were carried out on one NVIDIA GEFORCE RTX 4080 GPU. All images were resized into the resolution of 256 × 128. We chose Adam as the optimizer to optimize the framework with a weight decay of 0.0005. For data augmentation, we performed random erasing, cropping, and flipping.

Stage 1: Pre-training. We pre-trained three source datasets as described as before. We adopted the MEFE network with a GBR model as backbone networks and initialized them by parameters pre-trained on the ImageNet. The initialized parameters of each expert branch were the same, and they were updated during training. The batch size of all datasets was set to 64. We trained the model for 80 epochs with 100 iterations in an epoch and the learning rate was set to 0.00035, which decreased to 0.000035 at the 40th and 70th epochs.

Stage 2: Mutual mean teaching adaptation training. Our proposed model was initialized with the best parameters of the pre-trained model. We set the batch size to 12 using Market and Duke as the target domain, including 36 images for source domains and 12 images from target domain, and set the batch size to 9 using MSMT17 as the target domain. The total training stage took 120 epochs, and the learning rate was fixed to 0.00035. In each epoch, we conducted mini-batch DBSCAN clustering for all target datasets. In the experiments, the loss weights were initialized to $\varsigma_{id}^{t}=0.5$ and $\varsigma_{tri}^{t}=0.8$, and the ensembling momentum α was set to 0.999.

### 4.3. Comparison with State of the Art

To prove the superiority of our method, we compared our method with the state of the arts on four domain adaptation tasks: Market to Duke, Duke to Market, Market to MSMT and Duke to MSMT, as shown in Table 1. From the results, we found that our method (MEFE-MIBN + GBR-GAT&GCN) outperformed other UDA person re-ID methods by at least 10.3%/8.4% and 11.5%/6.3% in mAP/top-1 from Market to Duke and from Duke to Market, respectively. The performance of our method even surpasses some fully supervised methods. The results show our model’s superiority in UDA person re-ID tasks. Nevertheless, some methods obtain better results on mAP and top-1 using MSMT17 as the target domain than ours. The main reason is that our method utilizes both the diversity and consistency between different domains to constrain models in the manner of mutual learning, leading to higher computational complexity and a smaller batch size on one NVIDIA GEFORCE RTX 4080 GPU.

**Figure 3:**
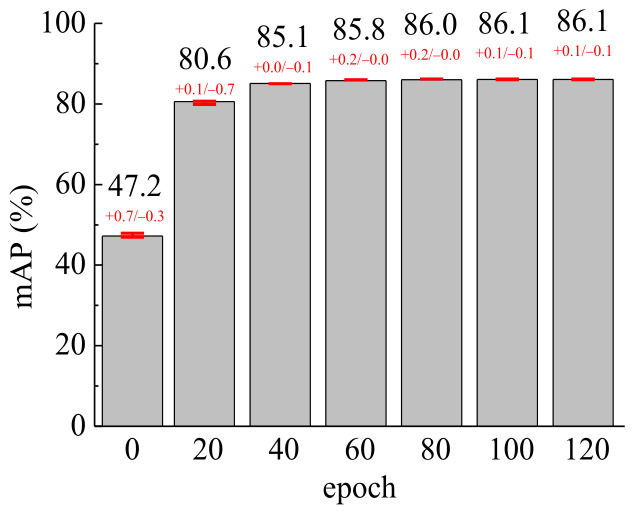
The mAP and confidence intervals from DukeMTMC-reID, CUHK03 and MSMT17 to Market1501 in 120 epochs.

To evaluate the stability and reproducibility of our method, we conducted three independent times, reporting the mAP and corresponding error ranges in Figure 3. The results show that, as training progresses, the mAP increases from 47.2% (epoch 0) to a stable 86.1% (epoch 100–120), while the error ranges gradually narrow from +0.7%/−0.3% to +0.1%/−0.1%. The consistently high performance with shrinking error ranges demonstrates that the reported improvements are robust, not due to random variation, confirming the effectiveness and reliability of our multi-source domain adaptation strategy.

### 4.4. Ablation Study

#### 4.4.1. Effectiveness of IBN in MEFE Network

The normalization layer in the MEFE network of the baseline is built via a single BN layer without a GBR module. The comparison experiment is conducted by applying an IBN layer in the MEFE network of the first three groups (conv2_x-conv4_x). The huge performance difference between the IBN-based MEFE and BN-based MEFE can be observed in Table 2, which indicates the efficiency of the IBN layers. Plugging the IBN into the MEFE module further improves the generalization ability. For the ResNet-50 backbone without GBR(GCN&GCN), the IBN-based MEFE improves model by 3.7% and 2.1% in mAP accuracy from Market to Duke and from Duke to Market. For the ResNet-50 backbone with GBR(GCN&GCN), we can observe similar improvements, which validate the capability of MEFE-IBN in generalization and discrimination for the target domain. Taking the results of GBR(GCN&GCN) as an example, the results of the model with GBR achieves 70.5% and 84.1% on mAP, which are larger than those without the GBR module. The results show the effectiveness and robustness of the GBR module.

#### 4.4.2. Effectiveness of DSI in MEFE Network

To investigate the effectiveness of DSI, we conducted ablation studies, as seen in Table 3. We trained the model with a different GBR module, the approaches with DSI performed better than the methods without DSI, especially from Duke to Marke. Specifically, with the model MEFE-IBN and without GBR, adding the DSI module increases the baseline by 0.3% and 0.6% in mAP and top-1 accuracy from Duke to Market. The results are similar after adding the GBR module in mAP and top-1 from Market to Duke. The IN layers eliminate style differences inevitably; the DSI module following the BN and IN layers can compensate for the missing domain-specific style information at the feature level.

#### 4.4.3. Effectiveness of GAT

We evaluate the GBR from two aspects: removing GBR and using GAT&GCN. As Table 4 shows, without IBN-based MEFE models, top-1 accuracy increases 0.1% compared to removing GBR when using GAT&GCN from Market to Duke and from Duke to Market. A similar improvement can be observed when adopting IBN-based MEFE models from Market to Duke and from Duke to Market. The effectiveness of the GBR(GAT&GCN) can be attributed to it enhancing the capability of integrating multiple domain center features adaptively.

#### 4.4.4. Effectiveness of Adaptive a_ij_ in GCN

To explore the effectiveness of adaptive weights in the adjacency matrix, we conducted ablation experiments, as shown in Table 5. Without adaptive represents the weight in the adjacency matrix of GCN being constant, which is assigned 1 for all the edges between the domain center nodes and feature nodes. With adaptive represents the value of the edges between domain center nodes and feature nodes calculated by Gaussian similarity function, and σ is the length scale parameter in the Gaussian similarity function. Firstly, almost experiments with adaptive weight perform better than the constant weight, which verifies the improvement in exploiting the implicit relation in the features. Secondly, compared with experiments with different values of σ, when the scale parameter σ is set to 2, we get better performance, which makes full use of the sample feature and domain center feature similarities.

#### 4.4.5. Effectiveness of Loss

We propose the mmd loss to reduce the distribution discrepancy between the source domain and target domain, improving the model’s generalization performance on the target domain. Furthermore, we introduce c-mmd loss to enforce the feature center of same-class samples to align closely between source and target domains, reducing intra-class discrepancy. Thus, integrating these losses can improve aggregated feature generalization. In order to verify the effectiveness of the proposed loss functions, we conducted ablation experiments on loss functions, as shown in Table 6. When L_mmd_ is added, mAP and top-1 accuracy increase by 0.1%/0.2% and 0.2%/0.1% from Market to Duke and from Duke to Market, respectively, demonstrating that L_mmd_ can help to reduce the distribution discrepancy between the source domain and target domain, improving the model’s generalization performance on the target domain. When L_c-mmd_ is added, the mAP and top-1 accuracy increase by 03%/0.4% and 0.1%/0.1% from Market to Duke and from Duke to Market, respectively, demonstrating that L_c-mmd_ can help to slightly enforce the feature center of same-class samples to align closely between source and target domains, reducing intra-class discrepancy.

We quantified the individual contribution of each loss via mAP and top-1 accuracy from DukeMTMC-reID, CUHK03 and MSMT17 to Market1501 in 60 epochs, as shown in Table 7. Removing L_sid-GBR_ causes mAP to drop by 0.6% and top-1 by 0.3%, confirming L_sid-GBR_ as a critical component for identity discrimination. Removing both L_sid-GBR_ and L_sid_ further degrades performance, underscoring their synergistic effect. Removing L_mmd_ + L_c-mmd_ causes top-1 accuracy to drop from 94.7% to 94.6%, while mAP marginally increases from 85.8% to 85.9%. This confirms that mmd losses, though not affecting mAP significantly, play a critical role in improving ranking precision at the top-1 position. Interestingly, ablating L_stri_ slightly improves mAP/top-1 (+0.3%/+0.4%), which may be caused by redundancy between L_stri_ and L_mmd_ + L_c-mmd_, whose combination imposes over-constraint.

#### 4.4.6. Effectiveness of Parameter Settings of Loss Function

**Figure 4:**
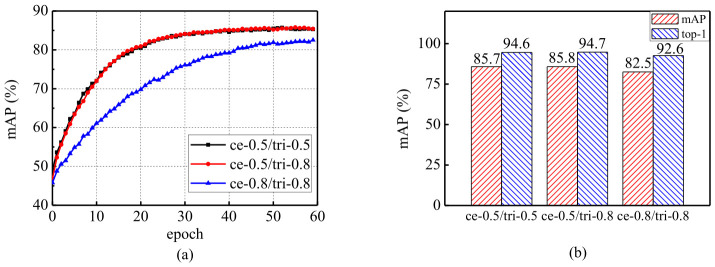
The mAP and loss comparisons of our method with different $\varsigma_{id}^{t}$ (id) and $\varsigma_{tri}^{t}$ (tri) from DukeMTMC-reID, CUHK03 and MSMT17 to Market1501 in 60 epochs. (**a**) The mAP curves of our method with different $\varsigma_{id}^{t}$ (id) and $\varsigma_{tri}^{t}$ (tri) in 60 epochs. (**b**) The maximum mAP and top-1 values of our method with different $\varsigma_{id}^{t}$ (id) and $\varsigma_{tri}^{t}$ (tri) in 60 epochs.

The effect of the two hyperparameters in the composite loss function on the model performance is evaluated in this section. The cross-entropy loss coefficient $\varsigma_{id}^{t}$ and triplet loss coefficient $\varsigma_{tri}^{t}$ are varied in the range of {0.5, 0.8} to investigate their impact on the training dynamics and final accuracy from DukeMTMC-reID, CUHK03 and MSMT17 to Market1501 in 60 epochs, as shown in Figure 4a,b.

As shown in Figure 4a, the training curves indicate that the convergence speed and peak mAP are primarily governed by $\varsigma_{id}^{t}$. A smaller $\varsigma_{id}^{t}$ = 0.5 yields faster convergence and higher asymptotic performance compared to $\varsigma_{id}^{t}$ = 0.8. Adjusting $\varsigma_{tri}^{t}$ within the studied range results in only marginal changes to the learning trajectory. The quantitative results in Figure 4b further confirm that the optimal configuration is $\varsigma_{id}^{t}$ = 0.5 and $\varsigma_{tri}^{t}$ = 0.8, which achieves the highest mAP of 85.8% and Rank-1 accuracy of 94.7%. These results demonstrate that a balanced weighting between classification and metric learning losses is critical for achieving robust feature representations in Re-ID tasks.

### 4.5. Visualization of Experimental Results

#### 4.5.1. Feature Maps Visualization Analysis

**Figure 5:**
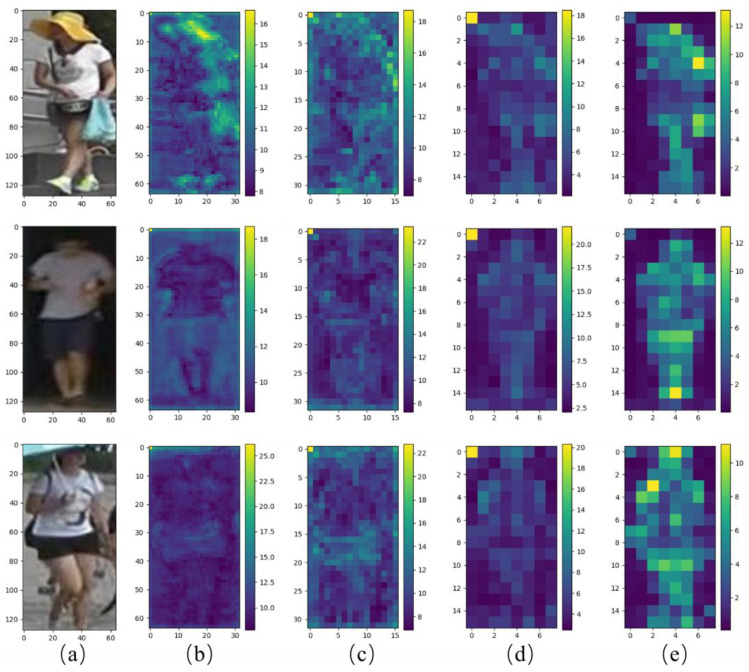
Visualization of the intermediate feature maps of the backbone. (**a**) The original image. (**b**) The intermediate feature maps of our method at layer 1. (**c**) The intermediate feature maps of our method at layer 2. (**d**) The intermediate feature maps of our method at layer 3. (**e**) The final feature map of the backbone model.

We visualize the intermediate feature maps of the backbone using our model with Market1501, as shown in Figure 5. Column (a) is the original image; columns (b), (c), and (d) are the intermediate feature maps of our method at layer 1, layer 2, and layer 3; and column (e) is the final feature map of the backbone model. Our method learns features from all the areas of the body and obtains a more comprehensive representation. This includes both pose and semantic information about the body. A comprehensive representation contributes to the elimination of perspective and pose differences.

#### 4.5.2. Top-List Visualization Analysis

**Figure 6:**
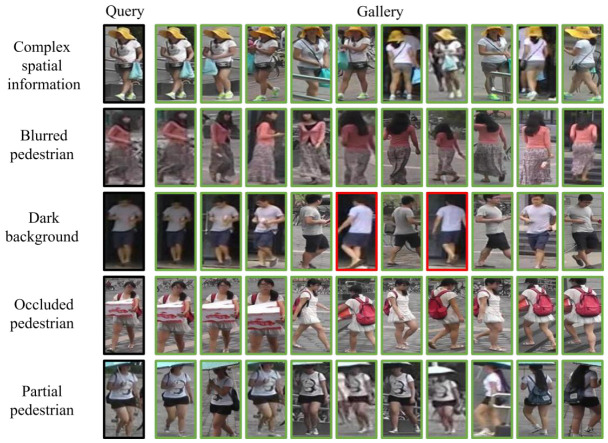
Retrieval results of our method. The image with a closed box is a query, the image with a green box is a correct retrieval result, and the image with a red box is an error retrieval result.

Some retrieval results are also visualized, as shown in Figure 6. The image with a closed box is a query, the image with a green box is a correct retrieval result, and the image with a red box is an error retrieval result. Figure 6 shows the retrieval results of our method. Regarding similarity, the matching algorithm selects the top 10 search results based on similarity scores. The retrieval results of our method include images from complex spatial information, blurred pedestrian images, occluded pedestrian images and partial pedestrian images. The results also demonstrate the effectiveness of our method. Only the fifth or seventh sequence in the third group was wrong, which once again proves the effectiveness of the pedestrian re-identification method proposed in this paper. By introducing multi-source domains and feeding them to the model for training, the model is able to learn ID-consistent representations from these diverse samples and achieve robustness with complex spatial information, blurred pedestrian images, occluded pedestrian images, partial pedestrian images, etc.

#### 4.5.3. T-SNE Visualization Analysis

**Figure 7:**
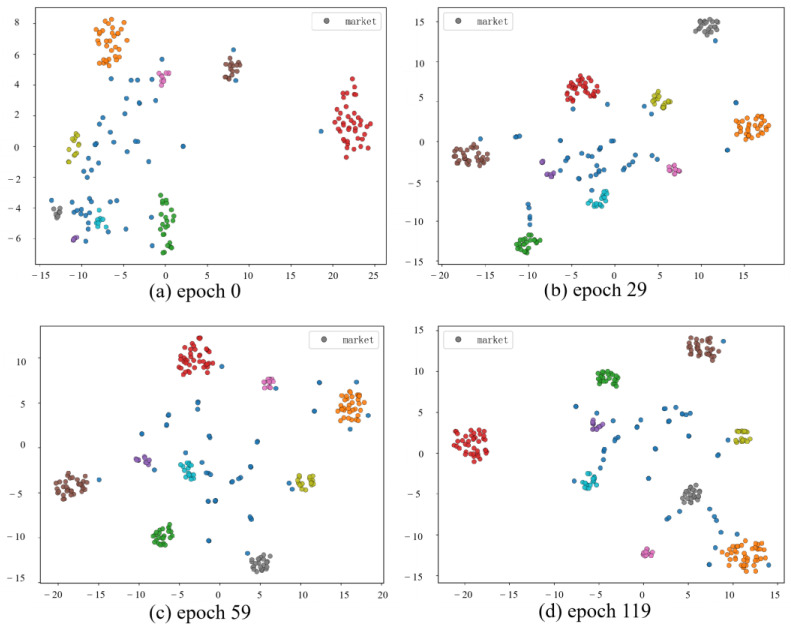
T-SNE [50] visualization of 10 random identities on Market. The features of the same identity (ID) are marked with the same color. For fair comparison, all subfigures have the same scale.

To visually demonstrate the effectiveness of the proposed multi-source domain adaptation strategy, we used t-SNE [50] to visualize the feature representations learned by our method at different training stages. Ten identities were randomly selected from the Market-1501 dataset, and their features were projected into a 2D space with color coding corresponding to identity labels, as shown in Figure 7.

At the initial stage (epoch 0), the features of different identities are severely mixed and poorly discriminative. A large number of samples from different classes overlap, and the intra-class distribution is scattered, indicating that the model has not yet learned discriminative identity information. As training progresses to epoch 29 and epoch 59, the feature distribution gradually changes: samples of the same identity begin to cluster tightly, while the inter-class separation gradually becomes clearer. Finally, at epoch 119, the features of each identity form compact and well-separated clusters, with significantly reduced intra-class variation and enhanced inter-class discriminability.

These results verify that by learning discriminative information from individual source domains, the model gradually improves its ability to handle unaligned hard samples. The progressive clustering and separation of features further confirm that our multi-source adaptation strategy effectively enhances the model’s discriminative power, thereby improving clustering accuracy and reducing intra-class variance.

## 5. Conclusions

In this work, we present a new approach combining a Mixture of Experts and a Graph-Based Relation module for unsupervised multi-source domain adaptation person re-identification. We develop a Mixture of Experts feature extraction network that adopts one general branch sharing all the parameters except for the normalization layers to learn domain-specific information. The combination of an IBN layer and BN layer is introduced into multiple experts to extract domain-invariant features across domains, and a domain-specific style information module is embedded into multiple experts to compensate for the missing domain-specific style information at the feature level. Meanwhile, we design a Graph-Based Relation module to integrate multiple domain features adaptively via cascading Graph Attention Networks and Graph Convolution Networks, and a Gaussian similarity function is utilized to exploit the implicit relation among features to construct the normalized relation matrix in the GBR module. Extensive experiments demonstrate that our method outperforms existing UDA person re-ID approaches on a large scale and achieves state-of-the-art results. Nevertheless, our method suffers from high computational complexity caused by multi-module stacking, limiting practical deployment. It also lacks robustness against complex real-world interference and relies heavily on sufficient source domain data. Future work will optimize lightweight network design, enhance environmental robustness, and reduce data dependence for better generalization.

## Figures and Tables

**Table 1:** Comparison with state-of-the-art methods on Market-1501 (M), DukeMTMC-reID (D), CUHK03 (C) and MSMT17 (MS).

Method		M(_C_MS)-to-D				D(_C_MS)-to-M			
		mAP	Top-1	Top-5	Top-10	mAP	Top-1	Top-5	Top-10
PDA-Net [42]	ICCV2019	45.1	63.2	77.0	82.5	47.6	75.2	86.3	90.2
SSG [27]	ICCV2019	53.4	73.0	80.6	83.2	58.3	80.0	90.0	92.4
HCT [43]	CVPR2020	50.7	69.6	83.4	87.4	50.7	80.0	91.6	95.2
MEB-Net [16]	ECCV2020	66.1	79.6	88.3	92.2	76.0	89.9	96.0	97.5
MMT(DBSCAN) [30]	ICLR2020	61.0	75.1	87.3	91.2	74.6	88.4	96.2	97.8
RLCC [44]	CVPR2021	69.2	83.2	**91.6**	**93.8**	77.7	90.8	96.3	97.5
PDA [45]	ICCV2021	70.8	**83.5**	-	-	83.4	94.2	-	-
MSUDA [32]	CVPR2021	68.9	82.1	90.4	93.0	86.0	**94.8**	97.9	98.6
SECRET-Joint [46]	AAAI2022	68.2	81.5	-	-	79.9	92.3	-	-
HCM [47]	TMM2023	67.9	82.3	90.2	92.8	79.0	91.8	96.7	97.7
IIDS [48]	TPAM2024	68.7	82.1	90.8	93.7	78.0	91.2	96.2	97.7
SAADA [49]	PRL2025	65.1	79.8	-	-	76.0	90.4	**-**	**-**
Ours (MEFE-MIBN + GBR-GAT&GCN)		**71.3**	**83.5**	**91.6**	93.5	**86.1**	94.7	**98.5**	**99.0**
		**M(_C_D)-to-MS**				**D(_C_M)-to-MS**			
SSG [27]	ICCV2019	13.2	31.6	-	49.6	13.3	32.2	-	51.2
MMT(DBSCAN) [30]	ICLR2020	18.3	41.2	50.3	55.6	17.1	41.8	50.2	55.1
RLCC [44]	CVPR2021	14.4	31.5	43.9	50.0	17.4	37.9	50.6	56.7
SECRET-Joint [46]	AAAI2022	25.4	51.2	-	-	**-**	-	**-**	**-**
HCM [47]	TMM2023	**26.9**	**59.6**	**70.1**	**74.3**	**26.9**	**59.6**	**70.1**	**74.3**
Ours (MEFE-MIBN + GBR-GAT&GCN)		26.4	53.8	66.4	71.6	26.4	53.8	66.4	71.6

The best outcomes are indicated in bold.

**Table 2:** Ablation studies on MEFE-IBN and GBR-GCN&GCN. Models are trained with three datasets except the target dataset; the datasets include Market-1501 (M), DukeMTMC-reID (D), CUHK03 (C) and MSMT17 (MS).

Base	MEFE-IBN	GBR-GCN&GCN	M(_C_MS)-to-D		D(_C_MS)-to-M	
			mAP	Top-1	mAP	Top-1
ResNet-50	×	×	66.6	80.3	81.9	93.2
ResNet-50	×	√	66.1	79.7	82.5	93.1
ResNet-50	√	×	70.3	83.0	84.0	93.3
ResNet-50	√	√	**70.5**	**83.5**	**84.1**	**93.7**

The best outcomes are indicated in bold.

**Table 3:** Ablation studies on DSI in MEFE. Models are trained with three datasets except the target dataset; the datasets include Market-1501 (M), DukeMTMC-reID (D), CUHK03 (C) and MSMT17 (MS).

Base	MEFE-IBN	MEFE-DSI	GBR-GAT&GCN	M(_C_MS)-to-D		D(_C_MS)-to-M	
				mAP	Top-1	mAP	Top-1
ResNet-50	×	×	×	66.6	80.3	81.9	93.2
ResNet-50	√	×	×	70.3	83.0	84.0	93.3
ResNet-50	√	√	×	70.4	83.0	84.3	93.9
ResNet-50	√	×	√	70.6	83.3	83.8	93.4
ResNet-50	√	√	√	70.5	83.3	84.1	94.1

**Table 4:** Ablation studies on GBR-GAT&GCN. Models are trained with three datasets except the target dataset; the datasets include Market-1501 (M), DukeMTMC-reID (D), CUHK03 (C) and MSMT17 (MS).

Base	MEFE-IBN	GBR-GAT&GCN	M(_C_MS)-to-D		D(_C_MS)-to-M	
			mAP	Top-1	mAP	Top-1
ResNet-50	×	×	66.6	80.3	81.9	93.2
ResNet-50	×	√	66.1	80.4	82.7	93.3
ResNet-50	√	×	70.3	83.0	84.0	93.3
ResNet-50	√	√	70.6	83.3	83.8	93.4

**Table 5:** Ablation studies on adaptive a_ij_ in GCN. Models are trained with three datasets except the target dataset; the datasets include Market-1501 (M), DukeMTMC-reID (D), CUHK03 (C) and MSMT17 (MS).

Base	Adaptive	σ	M(_C_MS)-to-D		D(_C_MS)-to-M	
			mAP	Top-1	mAP	Top-1
ResNet-50	×	×	70.6	83.3	83.8	93.4
ResNet-50	√	1	70.5	82.9	84.0	93.6
ResNet-50	√	2	70.6	83.0	84.3	94.1
ResNet-50	√	4	70.9	83.2	84.1	93.6

**Table 6:** Ablation studies on mmd loss function. Models are trained with three datasets except the target dataset; the datasets include Market-1501 (M), DukeMTMC-reID (D), CUHK03 (C) and MSMT17 (MS).

Base	L_mmd_	L_c-mmd_	M(_C_MS)-to-D		D(_C_MS)-to-M	
			mAP	Top-1	mAP	Top-1
ResNet-50	×	×	71.0	83.1	86.0	94.6
ResNet-50	√	×	71.1	83.3	86.2	94.7
ResNet-50	√	√	71.3	83.5	86.1	94.7

**Table 7:** Ablation studies on loss function. Models are trained with three datasets except the target dataset; the datasets include Market-1501 (M), DukeMTMC-reID (D), CUHK03 (C) and MSMT17 (MS).

Base	L_sid_	L_sid-GBR_	L_stri_	L_mmd_ + L_c-mmd_	Time	D(_C_MS)-to-M	
						mAP	Top-1
ResNet-50	√	×	√	√	9 h 8 min	85.2	94.4
ResNet-50	×	×	√	√	9 h 6 min	84.8	94.1
ResNet-50	√	√	√	×	9 h 1 min	85.9	94.6
ResNet-50	√	√	×	√	9 h 1 min	86.1	95.1
ResNet-50	√	√	√	√	9 h 6 min	85.8	94.7

## Data Availability

The experiment was conducted on three mainstream Re-ID datasets including Market-1501, DukeMTMC-reID and MSMT17. Market-1501: https://www.cv-foundation.org/openaccess/content_iccv_2015/papers/Zheng_Scalable_Person_Re-Identification_ICCV_2015_paper.pdf (accessed on 12 May 2026). DukeMTMC-reID: https://arxiv.org/abs/1609.01775 (accessed on 12 May 2026). MSMT17: https://arxiv.org/abs/1711.08565 (accessed on 12 May 2026).

## Abbreviations

The following abbreviations are used in this manuscript:

re-ID	person re-identification
UDA	unsupervised domain adaptation
MEFE	Mixture of Experts Feature Extraction
GBR	Graph-Based Relation
DSI	Domain-Specific Style Information
GAT	Graph Attention Networks
GCN	Graph Convolution Networks
mmd	maximum mean discrepancy
c-mmd	center maximum mean discrepancy
MoE	Mixture of Experts
BN	batch normalization
IN	instance normalization
MIBN	Mixture of Instance Normalization and Batch Normalization
IBN-a	Instance Batch Normalization type-a
DG	domain generalizable
GAN	Generative Adversarial Network
